# Cotton a Source of Error in Wassermann Test

**Published:** 1914-06

**Authors:** 


**Cotton a Source of Error in Wassermann Test.** H. Langer, Deutsche Med. Woch., Feb. 5, 1914, has found that the fat-like bodies absorbed from cotton, may give a positive or anti-complementary reaction. (Note. We have published from time to time, various reports showing that the Wassermann test may have a plu minus error of approximately 30%. Pending a thorough review of the evidence, the conclusion seems to be justified that only distinctly positive or negative tests should be relied upon and that the test is not one to be trusted to an inexpert observer simply following a laboratory manual).

